# Mitochondrial metabolic remodeling and blood–brain barrier dysfunction after repetitive mild traumatic brain injury

**DOI:** 10.3389/fnmol.2026.1852115

**Published:** 2026-10-16

**Authors:** Lingjuan Gao, Xincheng Xue, Xianhui Deng, Yu Xu, Heng Gao

**Affiliations:** 1 Department of Neurosurgery, Jiangyin Clinical College of Xuzhou Medical University, Jiangyin, Jiangsu, China; 2 Department of Neonatology, Jiangyin People’s Hospital, Jiangyin, Jiangsu, China

**Keywords:** blood–brain barrier, chronic traumatic encephalopathy, mitochondrial dysfunction, neurodegenerative diseases, repetitive mild traumatic brain injury

## Abstract

Repetitive mild traumatic brain injury (rmTBI) may arise when concussive or subconcussive impacts recur before physiological recovery is complete. Mitochondrial dysfunction and blood–brain barrier (BBB) impairment are shared features across the traumatic brain injury (TBI) spectrum; in rmTBI, repetition may alter their magnitude, duration, and temporal coupling. This focused narrative review evaluates evidence that mitochondrial metabolic remodeling contributes to neurovascular-unit dysfunction after rmTBI. We discuss energy failure, mitochondrial reactive oxygen species, succinate, fumarate, alpha-ketoglutarate, NAD + depletion, and lactate as candidate links between metabolic stress and BBB injury. Direct rmTBI evidence is prioritized, while single-TBI studies are used as mechanistic context when rmTBI data are limited. Available studies support a model in which recurrent metabolic stress, redox imbalance, inflammatory signaling, and impaired mitochondrial quality control converge on endothelial and multicellular neurovascular-unit dysfunction. Metabolism-directed interventions are promising but require validation across rigorously defined rmTBI paradigms and clinical cohorts.

## 1 Introduction

Repetitive mild traumatic brain injury (rmTBI) refers to two or more mild injuries, concussions, or subconcussive impacts occurring over intervals that may range from hours to years (Baskin et al., 2023; Howlett et al., 2022; Feinberg et al., 2023; Braun et al., 2024; Ratliff et al., 2020; Juan et al., 2022; Powell et al., 2023; McAllister and McCrea, 2017; Pierre et al., 2021). It affects athletes, military personnel, and people exposed to interpersonal violence. A central concern is that a subsequent impact can occur during incomplete recovery, potentially extending metabolic and neurovascular dysfunction. rmTBI has been associated with persistent cognitive symptoms and with neuropathological features relevant to chronic traumatic encephalopathy and other neurodegenerative disorders (Moraga-Amaro et al., 2025; Ojo et al., 2018; Ojo et al., 2019; Morin et al., 2020; Hiskens et al., 2023; McKee et al., 2023; Kirsch et al., 2023; Donison et al., 2025; Rosen et al., 2023; McKee et al., 2023; Powell et al., 2020; Lusardi et al., 2021; Guilhaume-Correa et al., 2025; Bang et al., 2016; Mohan et al., 2025).

Mitochondrial dysfunction, oxidative stress, neuroinflammation, and BBB disruption are not unique to rmTBI; they occur after single mild, moderate, and severe TBI. The rationale for this review is therefore not to claim a wholly distinct rmTBI mechanism. Rather, repeated impacts may modify the intensity, persistence, and coupling of shared secondary-injury processes. BBB dysfunction can permit entry of serum proteins and peripheral immune mediators, disrupt the tightly regulated neural microenvironment, and impair neurovascular coupling (Greene et al., 2026; Chagnot and Montagne, 2025; Menaceur et al., 2021; Mäe et al., 2021; Tang et al., 2023; Meng et al., 2023; Koh et al., 2025; Kadry et al., 2020; Abrahamson and Ikonomovic, 2020; Joseph, 2024; Uzunalli et al., 2021; Mantash et al., 2025; Sweeney et al., 2018).

This review focuses on the relationship between mitochondrial metabolic remodeling and BBB dysfunction within the neurovascular unit. It asks three linked questions: which metabolic changes are reported after rmTBI; by what mechanisms could these changes affect endothelial cells, pericytes, astrocytes, microglia, and neurons; and which metabolism-directed interventions merit further testing. We explicitly distinguish direct rmTBI observations from evidence derived from single-TBI models, which is used as mechanistic context rather than presented as rmTBI-specific proof.

Compared with earlier reviews of neurometabolic indicators in rmTBI (Kim et al., 2017), the present review concentrates on how selected mitochondrial metabolites and redox pathways may translate metabolic stress into a BBB phenotype. This evidence-tiered neurovascular-unit perspective is intended to generate testable hypotheses rather than imply that each proposed pathway has already been established in rmTBI.

## 2 Literature search strategy and scope

This article is a focused narrative review rather than a systematic review or meta-analysis. PubMed/MEDLINE, Web of Science, and Google Scholar were searched using combinations of “repetitive mild traumatic brain injury,” “repetitive TBI,” “concussion,” “blood–brain barrier,” “mitochondria,” “succinate,” “fumarate,” “alpha-ketoglutarate,” “NAD+,” “lactate,” “AMPK,” “oxidative stress,” and “neurovascular unit.” Reference lists were also screened (Salem et al., 2022, Mei et al., 2017, Lu et al., 2024). We prioritized reports that described impact number and interval, injury severity/model, species or clinical population, sampling compartment, and metabolic, BBB, inflammatory, or behavioral outcomes. Because no prospective PRISMA workflow was used, numerical screening counts are not reported.

Direct rmTBI studies were considered the primary evidence base. Single-TBI studies were retained only when they clarified a plausible mechanism or therapeutic approach, and are identified as contextual evidence. Clinical and animal evidence are interpreted separately because exposure ascertainment, injury timing, and outcome assessment differ substantially across study designs.

## 3 Immediate and cumulative effects of repetitive mild traumatic brain injury on the blood–brain barrier

### 3.1 Biomechanics and initial molecular events

The core injury mechanism of rmTBI arises from shear and stretching forces generated during head acceleration-deceleration or rotational movements (Donison et al., 2025). Unlike single severe traumatic brain injury, the biomechanical load in rmTBI is predominantly concentrated at the microvascular and axonal interfaces (Lauck et al., 2022). Owing to the differences in density and stiffness between cerebral blood vessels and the surrounding brain parenchyma, shear forces preferentially act on tight junctions (e.g., Claudin-5, Occludin) between endothelial cells and on the basement membrane, compromising the structural basis for vasogenic edema (Karakaya et al., 2026; Li et al., 2025; Gowrikumar et al., 2025). Concurrently, stretching forces directly disrupt the cytoskeleton (microtubules and microfilaments), induce axonal transport impairment, and activate mechanosensitive ion channels (e.g., Piezo1), triggering early calcium influx (Heyburn et al., 2023; Heyburn et al., 2019; Guo et al., 2024; Maneshi et al., 2018).

### 3.2 Excitotoxicity ionic imbalance and energy crisis

Mechanical injury compromises the integrity of neuronal and astrocytic membranes, leading to massive glutamate release and excessive activation of NMDA receptors, thereby inducing excitotoxicity (Lu et al., 2021; Lira et al., 2021; Tehse and Taghibiglou, 2019). Intracellular calcium overload further disrupts mitochondrial oxidative phosphorylation and precipitates an energy crisis (Bakaeva et al., 2022). Studies from both domestic and international sources indicate that mitochondrial dysfunction is particularly prominent in rmTBI: on one hand, abnormal accumulation of metabolic intermediates such as succinate exacerbates oxidative stress through reverse electron transport (Acosta et al., 2023; Feng et al., 2021; Allen et al., 2023; Jun et al., 2025); on the other hand, depletion of NAD^+^ suppresses DNA repair and SIRT1-mediated BBB protective pathways (Maynard et al., 2020; Jiang et al., 2023). This dual role of “upstream trigger” and “downstream amplifier” underlies the insidious and cumulative nature of pathological progression in rmTBI.

### 3.3 BBB dysfunction detection and biomarkers

The clinical manifestations of blood–brain barrier (BBB) disruption following rmTBI include paroxysmal headache, cognitive fluctuations, and increased susceptibility to secondary insults (Abrahamson and Ikonomovic, 2020; Hunter et al., 2019). Owing to the mild and intermittent nature of the injury, conventional neuroimaging often yields negative findings. Serum biomarkers currently serve as a critical tool for screening: (1) S100B: Originating from astrocytes, its low molecular weight allows leakage into the periphery even with mild increases in BBB permeability, offering high sensitivity but limited specificity (Jeon et al., 2025; Janigro et al., 2022; Gayger-Dias et al., 2023). (2) GFAP: Predominantly located in astrocytic endfeet, elevated serum levels of GFAP are closely associated with loss of BBB integrity and damage to the glial limitans, providing superior specificity compared to S100B (Janigro et al., 2022; Koerbel et al., 2024; Lafrenaye et al., 2020).

Imaging assessment has shifted from qualitative to quantitative approaches. Dynamic contrast-enhanced magnetic resonance imaging (DCE-MRI), utilizing pharmacokinetic models (e.g., Patlak or Tofts models), enables non-invasive quantification of regional increases in BBB permeability through calculation of the volume transfer constant (K_trans_) (Lee et al., 2024).

### 3.4 Consequences of BBB dysfunction

#### 3.4.1 Vasogenic cerebral edema

Disruption of the BBB allows plasma components such as albumin to enter the brain parenchyma, elevating tissue hydrostatic pressure and resulting in vasogenic edema (Zuo et al., 2025; Zhang et al., 2024; van Vliet et al., 2020). Unlike cytotoxic edema, this type of edema is more prominent in rmTBI and, owing to the direct toxicity of albumin to astrocytes, can induce reactive gliosis (Jia et al., 2025; Shandra et al., 2019; Zhang B. et al., 2025).

#### 3.4.2 Peripheral immune-cell infiltration and neuroinflammation

Peripheral myeloid cells, particularly pro-inflammatory phenotype macrophages, infiltrate the brain parenchyma through the damaged BBB and release inflammatory cytokines such as IL-1β and TNF-α (Huang et al., 2021; Hillmer et al., 2023). Concurrently, the release of damage-associated molecular patterns (DAMPs), including high mobility group box 1 (HMGB1), activates microglia, establishing a bidirectional crosstalk between the central and peripheral immune systems (Krämer et al., 2022). Of note, rmTBI often involves an “on–off” pattern of recurrent inflammatory stimulation, which predisposes microglia to an immune-primed state, ultimately contributing to long-term neurodegeneration (Cui et al., 2025).

#### 3.4.3 Entry of blood-derived neurotoxic mediators

Following the loss of BBB integrity, blood-derived substances such as thrombin, fibrinogen, and hemoglobin degradation products (e.g., heme) enter the brain parenchyma, directly inducing neuronal apoptosis and synaptic damage (Tian et al., 2023; Zafar et al., 2025; Dong et al., 2026). In addition, impaired glutamate clearance and diminished potassium buffering capacity further disrupt the homeostasis of the neuronal microenvironment, exacerbating synaptic dysfunction and neural network disconnection (Boyko et al., 2023; Ji et al., 2023; Woodman et al., 2023).

Repeated mechanical stress may establish persistent low-grade neuroinflammatory signaling after rmTBI. The causal contribution of BBB disruption to this process is likely bidirectional, but its magnitude and time course should be resolved in longitudinal, model-specific studies (Figure 1).

**Figure 1:**
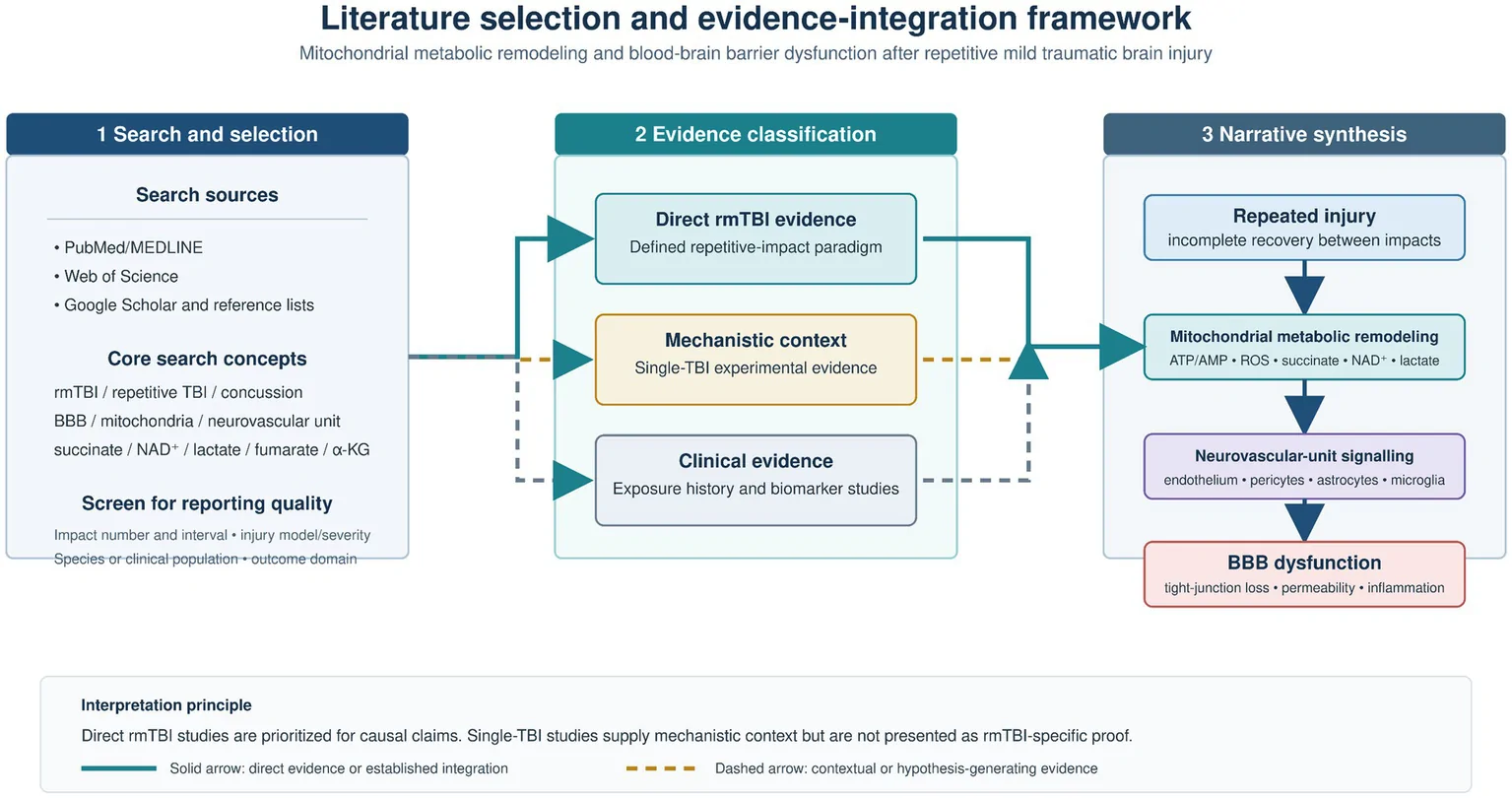
Literature selection and evidence integration framework for a focused narrative review of mitochondrial metabolic remodeling and BBB dysfunction after mTBl.

## 4 Mitochondrial dysfunction and metabolic remodeling after rmTBI

Although rmTBI does not cause the immediate, extensive tissue necrosis typically seen in a single severe traumatic brain injury, its cumulative effects, driven by repetitive mechanical stress, impose a sustained and insidious insult on mitochondria—the “energy hub” within neurons and vascular endothelial cells. An increasing body of research has revealed that mitochondrial dysfunction following rmTBI is not merely a matter of energy depletion, but rather a complex pathological process involving structural damage, dynamic imbalance, and profound remodeling of the metabolite profile (Kim et al., 2017; Acosta et al., 2023; Allen et al., 2023). This process constitutes the core driver of the transition from acute injury to chronic neurodegeneration.

### 4.1 Structural and bioenergetic dysfunction

In rmTBI, repetitive shear and stretching forces are directly transmitted to the intracellular environment, leading to physical disruption of the mitochondrial membrane and disorganization of cristae architecture. The influx of calcium ions activated by mechanical forces induces mitochondrial calcium overload (Kulbe and Hall, 2017). Excessive calcium uptake via the mitochondrial calcium uniporter (MCU) triggers sustained opening of the mitochondrial permeability transition pore (mPTP) (Donison et al., 2025). mPTP opening results in membrane potential collapse, uncoupling of the respiratory chain, and release of apoptotic factors such as cytochrome c, thereby initiating mitochondria-mediated apoptosis (Wang et al., 2022a).

Concurrently, the function of electron transport chain (ETC) complexes is significantly suppressed. Studies have confirmed decreased activity of Complex I and Complex IV in the cerebral cortex and hippocampus following rmTBI, leading to increased electron leakage and reduced ATP synthesis (Saha et al., 2022; Khan et al., 2023). The ATP/ADP/AMP ratio becomes severely imbalanced, and phosphocreatine, as an immediate energy buffer, is rapidly depleted, placing neurons in an “energy-vulnerable” state when responding to physiological activities (Mahajan et al., 2025).

Furthermore, the balance of mitochondrial dynamics is disrupted. Under physiological conditions, mitochondria maintain network homeostasis through fusion (regulated by Mfn1/2 and Opa1) and fission (regulated by Drp1 and Fis1) (Sidarala et al., 2022; Tábara et al., 2025). Following rmTBI, mitophagy pathways are compromised, resulting in impaired clearance of damaged mitochondria; the accumulation of dysfunctional mitochondria within cells releases abundant DAMPs (Karakaya et al., 2024).

### 4.2 Alterations in key mitochondrial metabolites

Mitochondrial dysfunction induced by rmTBI triggers systemic remodeling of the metabolite profile; these metabolic alterations act not merely as “consequences,” but rather as “signaling molecules” that in turn regulate neuroinflammation and BBB function.

#### 4.2.1 Energy metabolites and AMPK

Reduced ATP synthesis increases AMP relative to ATP and activates AMPK through upstream kinases including LKB1 and CaMKK2. Acute AMPK activation can be adaptive by conserving energy and supporting mitochondrial quality control. AMPK inhibits mTORC1 through TSC2 and Raptor and can thereby promote autophagy. Under persistent energy failure, however, dysregulated AMPK-mTOR signaling, inadequate autophagic flux, oxidative stress, and pro-apoptotic pathways may together contribute to injury. AMPK activation should therefore not be interpreted as intrinsically harmful or protective; its consequence depends on duration, cellular context, and the capacity to restore bioenergetic homeostasis (Muraleedharan and Dasgupta, 2022; Khayachi et al., 2024; Wani et al., 2021; Liu et al., 2008).

#### 4.2.2 Reactive oxygen species

Inhibition of electron transport chain complexes leads to increased electron leakage and excessive production of superoxide (O₂^−^) and hydrogen peroxide (H_2_O_2_), thereby triggering oxidative stress. Of note, in rmTBI, mitochondrial ROS not only act as mediators of damage but also drive the perpetuation of sterile inflammation through activation of the NLRP3 inflammasome and the NF-κB pathway (Yang et al., 2024; Bielanin et al., 2024; Mason et al., 2021; El-Gazar et al., 2024; Shaheen et al., 2021). Recent studies have indicated that the “pulsatile” release of mitochondrial ROS following repetitive mild injury serves as a critical initiating factor for microglial transition into an immune-primed state (Qian et al., 2024; Nguyen et al., 2023).

## 5 From mitochondrial metabolism to BBB dysfunction

Mitochondrial dysfunction after rmTBI may be recurrent rather than a single acute event. The proposed consequence is not that any individual metabolite invariably causes BBB failure, but that energy deficit, redox imbalance, and inflammatory signaling can act across the neurovascular unit. The following mechanisms are supported to different degrees by direct rmTBI observations and broader TBI literature.

### 5.1 Endothelial bioenergetics and tight-junction stability

Endothelial cells require ATP to maintain ion gradients, cytoskeletal organization, vesicular trafficking, and the localization of tight-junction proteins including claudin-5, occludin, and ZO-1. ATP depletion and mitochondrial ROS can therefore increase permeability and reduce recovery after injury. In rmTBI, repeated impacts may be particularly consequential when endothelial energy balance has not normalized; this temporal model remains to be tested directly.

### 5.2 Succinate NAD+ lactate fumarate and alpha-ketoglutarate

Succinate accumulation can increase mitochondrial ROS through reverse electron transport and can influence HIF-1alpha and inflammatory pathways. NAD+ depletion may limit sirtuin-dependent redox and repair responses. Lactate is both an energy substrate and a signaling molecule, with lactylation-related mechanisms emerging in neuroinjury. Fumarate should be considered separately from fumarate esters: endogenous fumarate may influence redox and electrophile-sensitive signaling, whereas dimethyl fumarate and monomethyl fumarate are pharmacological interventions. Alpha-ketoglutarate is a TCA-cycle intermediate and cofactor for dioxygenases, but direct causal evidence linking alpha-ketoglutarate changes to BBB dysfunction in rmTBI remains limited. These distinctions are now made explicit to avoid treating therapeutic pharmacology as proof of endogenous metabolite causality.

### 5.3 Inflammatory signaling and neurovascular-unit crosstalk

Mitochondrial ROS, succinate-associated signaling, and NAD + -sensitive pathways may converge on HIF-1alpha, NF-kB, and inflammasome activity. Downstream cytokines and VEGF can alter endothelial cytoskeletal tension and permeability. Pericyte contractility, astrocytic endfoot function, microglial activation, and neuronal energy demand can further modify the BBB phenotype. This multicellular framework explains why BBB disruption cannot be attributed to endothelial metabolism alone and highlights the need for cell-resolved, longitudinal rmTBI experiments (Shaheen et al., 2021; Balasubramanian et al., 2021; Zhou et al., 2024; Weng et al., 2025; Wu et al., 2021; Chang et al., 2025; Cao et al., 2025; Gulen et al., 2023; van Hameren et al., 2023; Yoon et al., 2025; Zhang Z. et al., 2025).

## 6 Metabolism directed therapeutic strategies

In the pathological evolution of rmTBI, systemic remodeling of mitochondrial metabolites has been established as a central hub driving BBB disruption and the perpetuation of neuroinflammation. Based on this, interventional strategies targeting mitochondrial metabolism are shifting from traditional “energy supplementation” toward “precise regulation of the metabolic signaling network.” This review will elaborate on the following aspects (see Table 1).

**Table 1:** Representative metabolism-directed interventions in experimental TBI.

Intervention	Target	Species and model	Dose and route	Reported outcome	Evidence level
MitoQ	Mitochondrial ROS	C57BL/6 mouse; three concussive injuries	5 mg/kg, intraperitoneal; twice weekly for 1 month	Reduced oxidative stress and chronic pathological outcomes	Direct rmTBI (Tabet et al., 2022)
Mdivi-1	Drp1-mediated fission	Experimental TBI	3 mg/kg, intraperitoneal	Reduced BBB disruption and cell death in the reported model	Single-TBI context (Wu et al., 2018)
Dimethyl fumarate	GSH/redox response	Experimental CCI TBI	80 mg/kg, oral	Neuroprotection linked to preservation of brain GSH; BBB benefit not established	Single-TBI context (Krämer et al., 2017)
Ethyl pyruvate	Metabolic and anti-inflammatory signaling	Rat CCI TBI	Study-specific regimen	Metabolic outcome in CCI; not direct rmTBI evidence	Single-TBI context (Golovachev et al., 2024)
NMN or NR	NAD + restoration	Experimental neuroinjury models	Study-specific regimen	Potential redox and mitochondrial support	Mechanistic context (Ning et al., 2024; Chai et al., 2025; Wang et al., 2022b; Thompson et al., 2024; She et al., 2025)

### 6.1 Substrate and cofactor supplementation

First, elevating NAD^+^ levels, as NAD^+^ is the sole substrate for the SIRT1/SIRT3 deacetylases and becomes significantly depleted following mitochondrial dysfunction (Ning et al., 2024; Chai et al., 2025). Supplementation with its precursors—nicotinamide mononucleotide (NMN) (Wang et al., 2022b) or nicotinamide riboside (NR) (Thompson et al., 2024)—effectively restores the NAD^+^ pool, activates SIRT1- and SIRT3-mediated mitochondrial antioxidant defense. Mitochondrial dysfunction occurs following rmTBI, and supplementation with NAD^+^ precursors may promote blood–brain barrier repair, thereby ameliorating rmTBI.

Second, replenishing energy substrates may be relevant. Pyruvate is transported into mitochondria and converted by pyruvate dehydrogenase to acetyl-CoA, which enters the TCA cycle; this process does not bypass respiratory-chain Complex I. The ethyl pyruvate study cited here used a controlled cortical impact model, not rmTBI. It is therefore discussed as single-TBI metabolic context and not as direct evidence of efficacy in rmTBI or of apoptosis inhibition in an rmTBI model (He et al., 2025; Hackett et al., 2023; Golovachev et al., 2024).

### 6.2 Mitochondria targeted antioxidant strategies

Mitochondria-targeted antioxidants may be more relevant than non-targeted agents when mitochondrial ROS is a dominant mechanism (Bai et al., 2023, Ghosh et al., 2024). MitoQ has direct rmTBI evidence: C57BL/6 mice subjected to three concussive head injuries received MitoQ (5 mg/kg, intraperitoneally, twice weekly for 1 month), with attenuation of oxidative stress and chronic pathological outcomes (Tabet et al., 2022). Evidence for other mitochondria-targeted antioxidants should not be extrapolated across diseases or injury paradigms without model-specific data.

### 6.3 Mitochondrial dynamics regulators

Excessive Drp1-mediated mitochondrial fission and impaired fusion may contribute to mitochondrial fragmentation after TBI. Mdivi-1 has shown effects on BBB disruption and cell death in experimental TBI, but this evidence is predominantly from single-TBI models and should be presented as mechanistic context rather than direct rmTBI evidence (Sridharan et al., 2024; Kulkarni et al., 2023; Qin et al., 2023; Wu et al., 2018; Zhang et al., 2021).

## 7 Conclusion

Mitochondrial dysfunction and BBB impairment are shared secondary-injury processes across TBI. In rmTBI, recurrent impacts may prolong or recouple energy stress, redox imbalance, inflammatory signaling, and neurovascular-unit dysfunction. Succinate, NAD+, lactate, fumarate, and alpha-ketoglutarate are useful candidates for testing this framework, but their causal evidence is not equivalent (Nengroo et al., 2025, Zhao et al., 2021, Xu et al., 2025, Ismail et al., 2020). Future work should report impact number and interval, injury severity, cell-specific metabolic measures, BBB endpoints, and longitudinal functional outcomes.

An evidence-tiered approach is important for translation. Studies should test whether timing of repeated impacts determines persistent metabolic and BBB phenotypes, and whether a given intervention restores a defined pathway rather than merely improving a broad injury score. This will help define the conditions under which metabolism-directed therapies are likely to be relevant to rmTBI.

At present, MitoQ provides direct preclinical rmTBI evidence, whereas NAD + precursor, ethyl pyruvate, dimethyl fumarate, and mitochondrial-dynamics approaches are supported mainly by single-TBI or related experimental models. These interventions should therefore be considered candidates for appropriately powered rmTBI studies, not established treatments.
